# Remodulation of the tumor microenvironment by regulatory T cells

**DOI:** 10.1186/2051-1426-3-S2-P409

**Published:** 2015-11-04

**Authors:** Chang Liu, Maria Chikina, Creg J Workman, Dario AA Vignali

**Affiliations:** 1 Department of Immunology, University of Pittsburgh, Pittsburgh, PA, USA; 2 Computational and Systems Biology, University of Pittsburgh, Pittsburgh, PA, USA; 3 University of Pittsburgh, Pittsburgh, PA, USA

## 

The tumor microenvironment is a complex system, which is composed of various types of non-tumor cells including stromal fibroblasts, the blood and lymphatic vascular networks, the extracellular matrix and notably, the infiltrating immune cells. Regulatory T cells (T_reg_ cells) play a pivotal role in tumor malignant progression and contribute to the resistance of tumors to traditional anti-cancer therapies; however, the elimination of T_reg_ cells is not a clinically viable approach, given their crucial role in maintaining immune homeostasis and preventing autoimmunity. Our laboratory has recently reported that T_reg_-restricted genetic disruption of Neuropilin-1 (Nrp1) selectively induced destabilization of T_reg_ cells within the tumor microenvironment, leading to tumor clearance without inducing autoimmunity. Interestingly, despite of their dramatic tumor-suppressive function, Nrp1-deficient T_reg_ cells are present in tumor in a comparable manner (number and kinetics) to their wild type counterparts, which provides an invaluable research tool to interrogate the function of intratumoral T_reg_ cells without physically removing them and inducing systemic autoimmunity. In this study we aim to systematically investigate the cellular and molecular mediators as well as the underlying mechanisms of T_reg_-cell function within the tumor microenvironment using a systems biology approach. With the unique T_reg_-restrictive Nrp1-deficient mice tumor models combined with a multifaceted approach that consists of flow cytometry based immunophenotyping and large-scale transcriptomic profiling, our results indicated that T_reg_ cells act as an early key regulator of tumor immune infiltration and actively induce the global remodulation of the tumor immune transcriptome. Computational deconvolution analysis of the gene profiling data derived from mixed populations further predicted a list of critically modified targets that are regulated at the single cell level. The functional validation of these targets may provide mechanism(s) by which T_reg_ cells interplay with the tumor microenvironment to potentiate tumor growth. This may lead to the development of novel and selective cancer immunotherapies.

